# Single-Shot Imaging of Two-Wavelength Spatial Phase-Shifting Interferometry

**DOI:** 10.3390/s19235094

**Published:** 2019-11-21

**Authors:** Jun Woo Jeon, Ki-Nam Joo

**Affiliations:** 3D Optical Metrology Laboratory, Department of Photonic Engineering, Chosun University, 309 Pilmun-daero, Dong-gu, Gwangju 61452, Korea; j.junwoooo@gmail.com

**Keywords:** spatial phase shifting, one shot imaging, two-wavelength interferometry, polarization pixelated camera

## Abstract

In this investigation, we propose an effective method to measure 3D surface profiles of specimens with single-shot imaging. Based on the two-wavelength interferometric principle and spatial phase-shifting technique using a polarization pixelated camera, the proposed system can not only rapidly measure the phase, but also overcome the 2π-ambiguity problem of typical phase-shifting interferometry. The rough surface profile can be calculated by the visibility of the interference fringe and can compensate for the height discontinuity by phase jumps occurring in a fine height map. An inclined plane mirror and a step height specimen with 9 μm were used for the validation of capability of measuring continuously smooth surface and large step heights. The measurement results were in good agreement with the results of typical two-wavelength interferometry.

## 1. Introduction

Optical interferometry is beneficial to measure 3D surface profiles of specimens as one of the non-destructive measurement techniques [1]. Phase-shifting interferometry (PSI) has high precision and has been widely used in optical shop testing because of its robust and reliable measurement results even though it suffers from the well-known 2π-ambiguity problem caused by the phase jumps [2,3]. Low-coherence scanning interferometry (LCSI) is free from the 2π-ambiguity problem because of the low-coherence characteristic to localize the interference fringe, i.e., correlogram, which enables to precisely measure the surface profiles of step height specimens as well as smooth surface targets [4]. However, the main drawback of LCSI is the scanning procedure to obtain the image stack in order to find the peak positions of correlograms [5]. The software-based unwrapping is mainly used for smooth surfaces and it can provide lots of errors for discrete surfaces such as step height specimens. On the other hand, multi-wavelength interferometry has been approached with the techniques of wavelength scanning [6,7] and synthetic wavelength [8,9] in order to avoid 2π-ambiguity problems of PSI. Also, the interferometric principle is adopted to extract the phase with the numerical wave propagation and 2π-ambiguity problems are also overcome with a multi-wavelength interferometric scheme [10,11,12] and a rapid operation of phase-shifting techniques [13] in digital holography. However, they have the fundamental limitations of the measurement speed by scanning wavelength and temporal phase shifting.

A polarization-pixelated camera (PCAM) has recently been used to various research fields such as polarization imaging [14], photoelastic measurements [15] and 3D imaging [16,17,18,19,20,21] because its single image contains four kinds of different polarized sub-images. In 3D imaging especially, the PCAM enables to obtain four phase-shifted images at once and rapidly calculate the phase map without any mechanical or electrical moving parts for temporal phase shifting. This spatial phase-shifting capability of the PCAM for measuring 3D surface profiles was firstly introduced as dynamic interferometry [17] and has been applied to interferometry [17,18], microscopy [19], digital holography [20] and low-coherence scanning interferometry [21] although it still experiences the 2π-ambiguity. Recently, a color PCAM has been adopted to implement snapshot 3D surface profilometry with the multi-wavelength interferometric technique to eliminate the 2π-ambiguity problem [22,23]. The color PCAM consists of a polarizer and a Bayer color filter array, which enables extraction of the phases for three wavelengths at once. However, the structure of the color PCAM significantly lowers the lateral resolution and the imperfection of the color filters deteriorates the phase corresponding to each wavelength of the light in spite of the pixel interpolation and color calibration techniques. The measurement errors by the chromatic aberration and unexpected phase retardation of the optical components including wavelength-dependent waveplates are also challenging to overcome. Furthermore, the equivalent wavelengths based on three wavelengths and even their cascaded equivalent wavelengths are not sufficiently long to measure the surface profile of various stepped specimens because of the large wavelength differences.

In this investigation, we describe effective single-shot spatial phase-shifting interferometry. Instead of three-wavelength interferometric configuration using a color PCAM with much effort, two-wavelength interferometry with a single image obtained by a monochromatic PCAM is proposed and experimentally verified.

## 2. Principle of Spatial Phase-Shifting Interferometry Using A Single Image of Polarization-Pixelated Camera (PCAM)

### 2.1. Spatial Phase-Shifting Interferometry Using A PCAM

**Figure 1:**
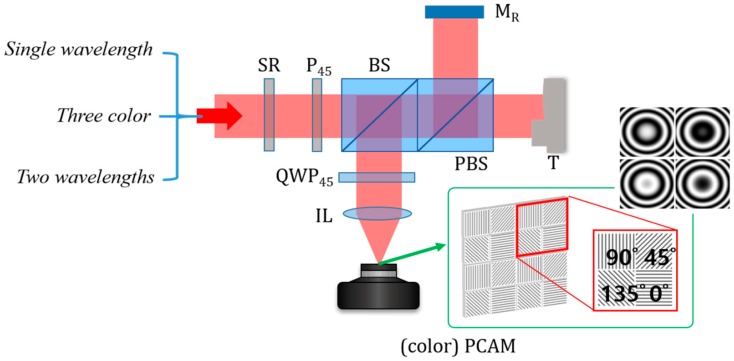
Optical configuration of spatial phase shifting interferometry using a polarization-pixelated camera (PCAM); SR, speckle reducer; P_45_, 45° rotated polarizer; BS, beam splitter; PBS, polarizing beam splitter; QWP_45_, 45° rotated quarter waveplate; IL, imaging lens; M_R_, reference mirror; T, target; (color) PCAM, (color) polarization-pixelated camera.

Figure 1 shows the optical configuration of a compact design of spatial phase-shifting interferometry based on polarizing optical components. As a light source, a laser is used and the light is delivered by an optical fiber. In order to reduce the unwanted coherent noise such as speckle and diffraction patterns, a speckle reducer (SR), which consists of rotating a diffuser is applied and the polarization of light becomes 45° linearly polarized with a 45° rotated polarizer (P_45_). The polarized light incident to a polarizing beam splitter (PBS) after going through a beam splitter (BS) is divided into reference and measurement lights. The reference and measurement lights are reflected by a reference mirror (M_R_) and a target (T), respectively, and they are reflected by the BS and passes through a quarter wave plate (QWP_45_), of which fast axis is rotated as 45°. Then, two linearly polarized lights become two circularly polarized lights and detected by a PCAM. In the PCAM, each individual pixel has its own polarizer and these polarizers are oriented with 0°, 45°, 90° and 135° and repeated with two-pixel blocks as seen in the inset of Figure 1. By each polarizer in the PCAM, the reference and measurement lights can induce the interference and four kinds of phase-shifted interference fringes can be obtained at once. Subsequently, the phase map corresponding to the surface profile of the target can be obtained with single imaging.

Mathematically analyzing the operation of the system can be implemented and the initial linearly polarized light (*E_in_*) by the P_45_ can be described with the Jones vector as:

(1) $$E_{in}=E_{0}\left(\begin{array}{c} 1\\ 1 \end{array}\right),$$

where *E*_0_ indicates the amplitude of the light. Then, the reference (*E_r_*) and measurement (*E_m_*) lights divided by the PBS after reflecting off by M_R_ and T can be represented as:

(2) $$\begin{array}{l} E_{r}=\frac{E_{0}}{2}e^{j\varphi_{r}}\left(\begin{array}{c} 1\\ 0 \end{array}\right)\\ E_{m}=\frac{E_{0}}{2}e^{j\varphi_{m}}\left(\begin{array}{c} 0\\ 1 \end{array}\right) \end{array},$$

where *φ_r_* and *φ_m_* are the phases caused by the optical path lengths and *j* indicates the imaginary number as $\sqrt{-1}$. When *E_r_* and *E_m_* pass through QWP_45_, these two linearly polarized lights are converted as two circularly polarized lights as:

(3) $$\begin{array}{l} E_{r}=\frac{E_{0}}{4}e^{j\varphi_{r}}\left(\begin{array}{c} 1+j\\ 1-j \end{array}\right)\\ E_{m}=\frac{E_{0}}{4}e^{j\varphi_{m}}\left(\begin{array}{c} 1-j\\ 1+j \end{array}\right) \end{array},$$

Then, four kinds of rotated polarizers in the PCAM can generate the phase shifted interference fringes, respectively, as follows:

(4) $$\begin{array}{l} I_{0}={\left|E_{r,0}+E_{m,0}\right|}^{2}=A(1+\gamma \sin\varphi )\\ I_{45}={\left|E_{r,45}+E_{m,45}\right|}^{2}=A(1+\gamma \cos\varphi )\\ I_{90}={\left|E_{r,90}+E_{m,90}\right|}^{2}=A(1-\gamma \sin\varphi )\\ I_{135}={\left|E_{r,135}+E_{m,135}\right|}^{2}=A(1-\gamma \cos\varphi ) \end{array},$$

where *I*_0_, *I*_45_, *I*_90_ and *I*_135_ are the intensity detected by 4 different pixel sets of the PCAM, respectively, and *A* is the mean intensity of the interference fringe denoted as (*E*_0_^2^/4). *φ* means the phase difference between the reference and measurement lights as (*φ_m_* − *φ_r_*) and *γ* indicates the visibility of the interference fringe. Because the interference fringes in Equation (4) are shifted as 90°, the phase, *φ* can be extracted by the well-known 4-step phase shifting algorithm as [17]:

(5) $$\varphi =\varphi_{m}-\varphi_{r}=\tan^{-1}\left(\frac{I_{0}-I_{90}}{I_{45}-I_{135}}\right),$$

### 2.2. Three-Color Interferometry Using A Color PCAM

In monochromatic spatial phase shifting interferometry, the phase can be obtained by Equation (5), but the phase jumps by the 2π-ambiguity still restrict measurement of surface profiles if any kinds of height steps larger than the half wavelength are on the surfaces. The use of color PCAM and lasers with three different wavelengths can overcome the limitation of the system because the measurable maximum height can be extended when the equivalent wavelengths based on three wavelengths corresponding to red, green and blue colors are applied. In case of using two wavelengths, for instance, the equivalent wavelength (Λ_12_) is defined as [9].

(6) $$\Lambda_{12}=\frac{\lambda_{1}\lambda_{2}}{\left|\lambda_{1}-\lambda_{2}\right|},$$

where λ is the wavelength of the light when the equivalent phase (Φ_12_) is calculated as [8]:

(7) $$\Phi_{12}=\varphi_{1}-\varphi_{2},$$

Then, the measured height (*H*_12_) by the equivalent wavelength is calculated as [9]:

(8) $$H_{12}=\frac{\Lambda_{12}}{2}\frac{\Phi_{12}}{2\pi },$$

**Figure 2:**
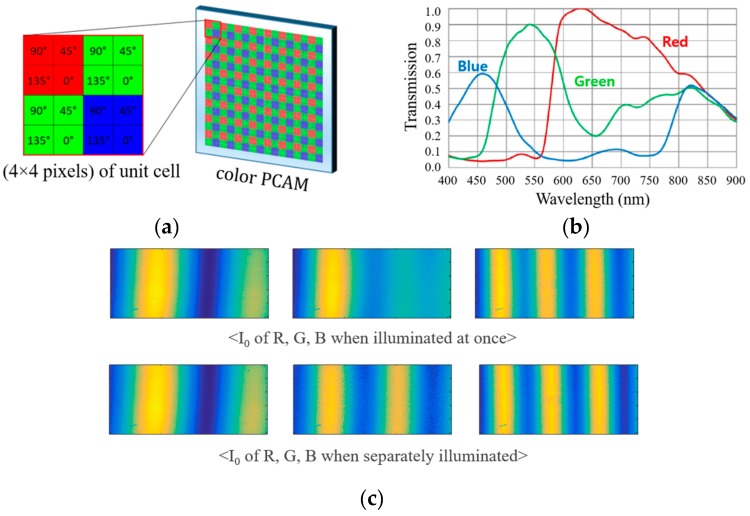
(**a**) Structure of a color PCAM and its unit cell; (**b**) transmission characteristics of color filter in the color PCAM; (**c**) color crosstalk in the interference fringes.

In this case, however, the height error is also increased by a factor of (Λ_12_/*λ*_1_) because the height error is the result of multiplication of the wavelength and phase error. Even though the phase error of the equivalent phase has the similar level of the original phase error, the height error can be magnified by the equivalent wavelength. To avoid this magnified phase error, the fringe order method, where Λ_12_ determines the order of phase jumps (*N*) only, can be used. Then, the height *H* is calculated as follows [9]:

(9) $$H=\frac{\lambda_{1}}{2}\left(\frac{\varphi_{1}}{2\pi }+N\right),$$

(10) $$N=\mathrm{int}\left[\frac{2H_{12}}{\lambda_{1}}\right],$$

where int[x] provides the integer of x only. By using this fringe order method, high precision measurements can be achieved even though the measurement range is longer than the half wavelength. In three wavelength interferometry, this technique can be further extended by cascading the equivalent wavelengths using Λ_12_ and Λ_23_ as [21]:

(11) $$\Lambda_{123}=\frac{\Lambda_{12}\Lambda_{23}}{\left|\Lambda_{12}-\Lambda_{23}\right|},$$

with its phase (Φ_123_) derived in a similar manner. However, the color PCAM is operated by the combination of the polarizer and the color filter arrays as illustrated in Figure 2a and the unit cell (4 × 4 pixels) [23] to extract the same height information should be four times larger than that (2 × 2 pixels) of a monochromatic PCAM. This limitation of PCAM structure can induce the mismatch of the measuring point corresponding to each pixel and lowers the lateral resolution of the measurement system. Even though several techniques based on pixel interpolation can be applied [22,23], the effectiveness is fundamentally limited. Another important issue of the color PCAM is the imperfection of the color filter, which leads to the interference fringe mixing between three wavelengths. As shown in Figure 2b, each color filter used in the PCAM has a wide transmission spectrum and even the optical densities of the filters to prevent other colors are not so high. Because of these features of the color PCAM, especially, crosstalk among RGB signals as seen in Figure 2c, photon response non-uniformity, and polarizer extinction ratio nonuniformity should be calibrated with much effort in addition to chromatic aberration of the system. Furthermore, these calibrations may be performed repeatedly with various targets and optical configurations.

### 2.3. Single-Shot Imaging of Two-Wavelength Interferometry

Instead of using the color PCAM, a typical monochromatic PCAM has smaller unit cell (2 × 2 pixels) to alleviate the sacrifice of the lateral resolution. Also, its chromatic features do not have to be considered in the system if the light source is monochromatic or quasi-monochromatic. The only concern of the system is to avoid the 2π-ambiguity problem and we adopt two-wavelength interferometry, where the wavelengths of the laser sources are close to each other, and obtain a single image as the summation of interference fringes of two lights. In order to explain the operating principle of single shot two-wavelength interferometry, Equation (4) can be rewritten with two wavelengths (*λ*_1_ and *λ*_2_) as:

(12) $$\begin{array}{l} I_{0}={\left|E_{r,0}+E_{m,0}\right|}^{2}{}_{\lambda_{1}}+{\left|E_{r,0}+E_{m,0}\right|}^{2}{}_{\lambda_{2}}=A_{1}(1+\gamma_{1}\sin\varphi_{1})+A_{2}(1+\gamma_{2}\sin\varphi_{2})\\ I_{45}={\left|E_{r,45}+E_{m,45}\right|}^{2}{}_{\lambda_{1}}+{\left|E_{r,45}+E_{m,45}\right|}^{2}{}_{\lambda_{2}}=A_{1}(1+\gamma_{1}\cos\varphi_{1})+A_{2}(1+\gamma_{2}\cos\varphi_{2})\\ I_{90}={\left|E_{r,90}+E_{m,90}\right|}^{2}{}_{\lambda_{1}}+{\left|E_{r,90}+E_{m,90}\right|}^{2}{}_{\lambda_{2}}=A_{1}(1-\gamma_{1}\sin\varphi_{1})+A_{2}(1-\gamma_{2}\sin\varphi_{2})\\ I_{135}={\left|E_{r,135}+E_{m,135}\right|}^{2}{}_{\lambda_{1}}+{\left|E_{r,135}+E_{m,135}\right|}^{2}{}_{\lambda_{2}}=A_{1}(1-\gamma_{1}\cos\varphi_{1})+A_{2}(1-\gamma_{2}\cos\varphi_{2}) \end{array},$$

where 1 and 2 indicate the parameters related to *λ*_1_ and *λ*_2_. If *λ*_1_ and *λ*_2_ are close to each other, most of optical components including the PCAM provide almost the same optical characteristics. Moreover, two laser lights can be delivered to the interferometer by the same optical fiber. Then, *r*_1_ and *r*_2_ can be set as the same *r* based on the high temporal coherence of lasers and it can be preliminary determined by the reflectivities of the reference mirror and the target. Under the condition of the same optical powers of two lasers, Equation (12) can then be summarized by identities of trigonometric functions as:

(13) $$\begin{array}{l} I_{0}=A\left[1+(\gamma \cos\varphi_{rough})\sin\varphi_{fine}\right]\\ I_{45}=A\left[1+(\gamma \cos\varphi_{rough})\cos\varphi_{fine}\right]\\ I_{90}=A\left[1-(\gamma \cos\varphi_{rough})\sin\varphi_{fine}\right]\\ I_{135}=A\left[1-(\gamma \cos\varphi_{rough})\cos\varphi_{fine}\right] \end{array},$$

where *A* is denoted as (*A*_1_ + *A*_2_ = 2*A*_1_ = 2*A*_2_). *φ_fine_* and *φ_rough_* indicate (*φ*_1_ + *φ*_2_)/2 and (*φ*_1_ − *φ*_2_)/2, respectively. In this case, *φ_fine_* as the phase at an equivalent wavelength (Λ*_fine_* = 2*λ*_1_*λ*_2_/|*λ*_1_ + *λ*_2_|) is calculated by Equation (5). On the other hand, *φ_rough_* as the phase at an equivalent wavelength (Λ*_rough_* = 2*λ*_1_*λ*_2_/|*λ*_1_ − *λ*_2_|) can be also extracted as:

(14) $$\varphi_{rough}=\cos^{-1}\left[\frac{2}{\gamma }\sqrt{\frac{{\left(I_{0}-I_{90}\right)}^{2}+{\left(I_{45}-I_{135}\right)}^{2}}{{\left(I_{0}+I_{90}+I_{45}+I_{135}\right)}^{2}}}\right],$$

As the result, Λ*_rough_* becomes long wavelength and *φ_rough_* is used to determine the integer number of phase jumps of *φ_fine_* as exactly same as two wavelength interferometric principle because Λ*_fine_* indicates short wavelength with similar order of magnitude of *λ*_1_ and *λ*_2_. Consequently, the proposed technique can measure the 3D surface profile with a single image to minimize the chromatic features of the system, avoid significant lateral resolution decrease and increase the equivalent wavelength without any cascading calculations opposed to three-wavelength interferometry using a color PCAM. 

## 3. Results

**Figure 3:**
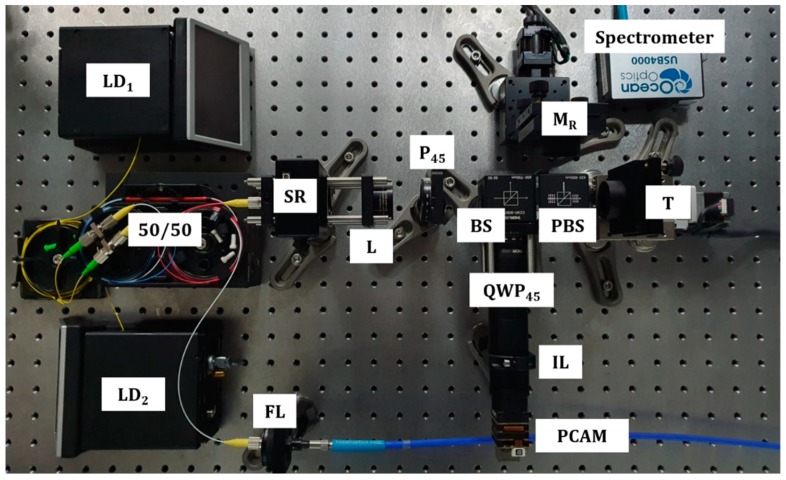
Photographs of the proposed system; LD, fiber Bragg grating laser diode, SR, speckle reducer; L, lens; P_45_, 45° rotated polarizer; BS, beam splitter; PBS, polarizing beam splitter; QWP_45_, 45° rotated quarter waveplate; IL, imaging lens; FL, focusing lens; M_R_, reference mirror; T, target; PCAM, polarization- pixelated camera.

In order to verify the measurement principle of the proposed system, feasibility tests were implemented with a plane mirror and a step-height specimen. As shown in Figure 3, as light sources, two fiber Bragg grating (FBG) laser diodes (BLD-633-14BF, BLD-641-14BF, NOLATECH, Moscow, Russia) at 633 nm (LD_1_) and 641 nm (LD_2_) were used for the stable single laser frequency sources and Λ*_fine_* and Λ*_rough_* were calculated as 637.0 nm and 101.4 μm, respectively. A PCAM (Blackfly^®^ S Polarization camera, FLIR, Wilsonville, OR, USA), which has (2448 × 2048) pixels and 3.45 μm pixel size, detected the whole interference fringes, divided into four phase shifted images with (1224 × 1024) pixels. The wavelengths of the sources were measured by a commercial spectrometer (USB4000, OceanOptics, Largo, FL, USA).

**Figure 4:**
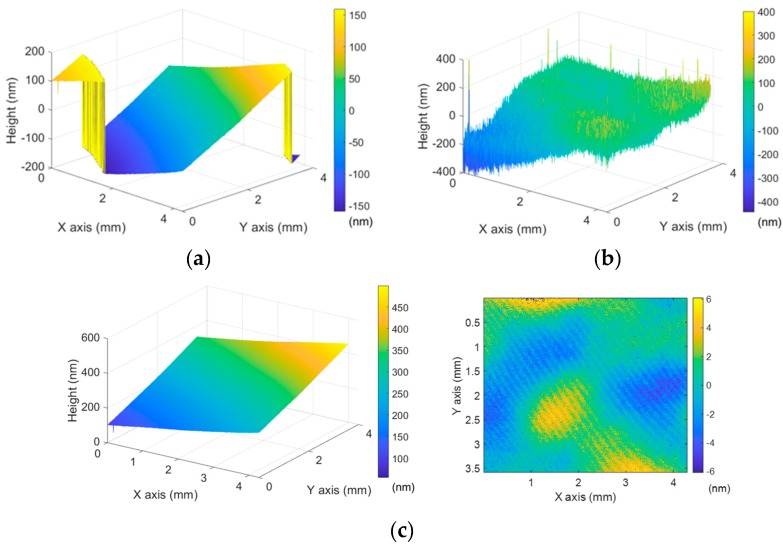
(**a**) Height map of the plane mirror by *φ_fine_* with height jumps; (**b**) height map by *φ_rough_*; (**c**) calibrated height map.

Figure 4a presents the height map with height jumps caused by the wrapped phase map of *φ_fine_* by the slope of the plane mirror surface. On the other hand, the height map with *φ_rough_* did not experience the 2π-ambiguity because of the long Λ*_rough_* as shown in Figure 4b and, finally, the height map of *φ_fine_* was calibrated by Equation (9) as illustrated in Figure 4c.

**Figure 5:**
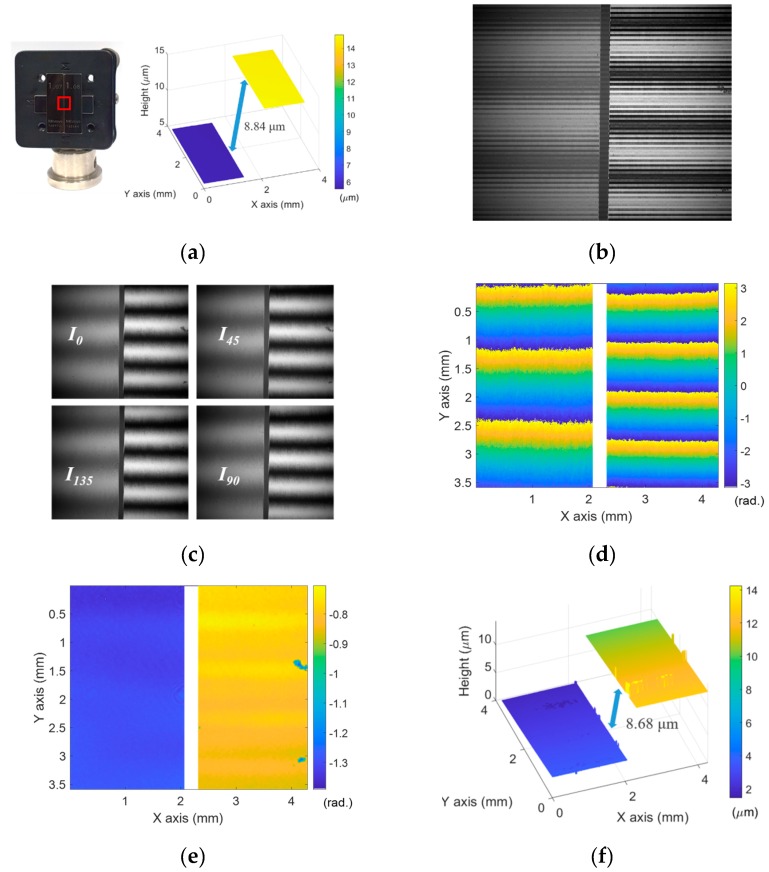
(**a**) Picture of the step height specimen and the measurement result of the step height specimen by low-coherence scanning interferometry (LCSI); (**b**) whole interference fringe of the PCAM; (**c**) four phase-shifted interference fringes; (**d**) wrapped phase map of *φ_fine_*; (**e**) *φ_rough_* obtained by Equation (14) from the visibility; (**f**) calibrated height map.

A step-height specimen was ready for further validation of capability of the proposed method to measure larger step heights. The specimen was constructed with two gauge blocks, which had slightly different heights, and the step height difference was approximately 10 μm less than Λ*_rough_*/4. Figure 5a shows the picture of the specimen and the measurement result by LCSI which has 8.84 μm step height. Figure 5b presents the whole interference fringe and it was divided into four phase-shifted images as seen in Figure 5c. Figure 5d shows the wrapped phase map of *φ_fine_* calculated by Equation (5) and *φ_rough_* was obtained by Equation (14) from the visibility of the interference fringes as shown in Figure 5e. Based on 2D phase unwrapping technique and Equation (10), the phase jumps of *φ_fine_* were compensated and the 3D surface profile of the specimen was reconstructed as presented in Figure 5f. As the measurement result, the mean value of the step height was 8.68 μm. It is noted that the interface edge areas of the gauge blocks were eliminated from the data because of low reflected intensity level of the light on the edge by the low numerical aperture of the system.

**Figure 6:**
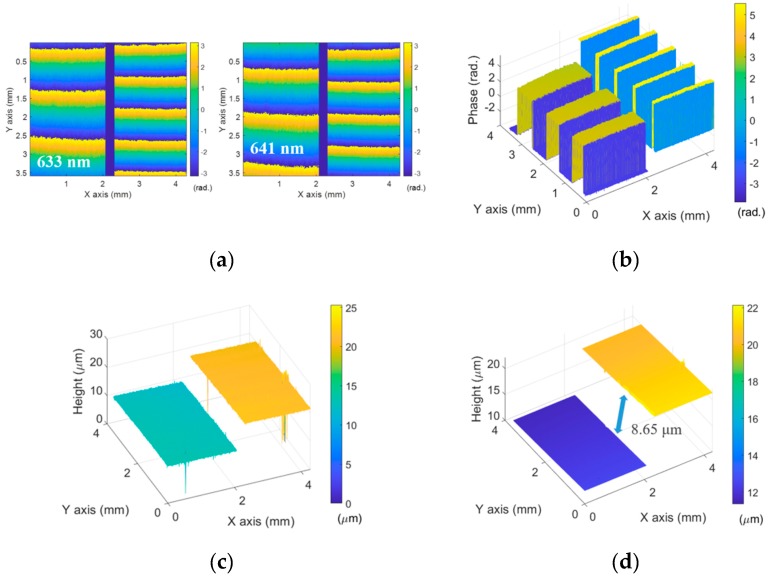
(**a**) Wrapped phase maps at 633 nm (*λ*_1_) and 641 nm(*λ*_2_); (**b**) equivalent phase map (Φ_12_); (**c**) height map by Φ_12_; (**d**) calibrated height map of the step height specimen.

For the comparison of the result, the same specimen was measured by the typical two-wavelength interferometric technique. Figure 6a shows the wrapped phase maps at 633 nm (*λ*_1_) and 641 nm (*λ*_2_) and the equivalent phase map (Φ_12_) was obtained by Equation (7) as illustrated in Figure 6b. In this case, the phase map still had phase jumps, but they were fixed under the condition of (−π < Φ_12_ < π) and the height map was obtained as Figure 6c. In the same manner, the phase jumps were compensated for and the 3D surface profile was obtained as seen Figure 6d. In this case, the step height was 8.65 μm.

As known in Figure 5f and Figure 6d, the proposed method has slight errors to fix the phase jumps and the height map has the specific portion of height jumps. This originated in the measurement error of the visibility, but most of the phase jumps were properly compensated for. For estimating system performance, the repeatability defined as a standard deviation of the mean step height values for 20 consecutive measurements, was calculated and it was 21.2 nm. In typical two-wavelength interferometry, it was 8.7 nm. This was mainly attributed to the compensation errors of the phase jumps as seen in Figure 5f.

## 4. Discussion

The main advantage of the proposed technique is the ability to obtain the 3D surface profile of the specimen at once without any significant lateral resolution reduction and chromatic features of the optical system. Opposed to the typical two-wavelength interferometry, a single image is only used to extract the rough and fine phase maps and overcome the 2π-ambiguity. The proposed technique can be also applied to rapidly recognize the phase objects based on the visibility and phase extraction. The proposed technique is fundamentally based on two-wavelength interferometry with equivalent wavelength. Therefore, the resolution and measurement uncertainty can be referred to the previous research works, which focus on the evaluation of the system performance. However, the determination of the rough surface with the long equivalent wavelength is distinguished from others because it was measured by the visibility of the interference fringe with the single image. The concern with using the visibility to obtain the rough surface profile is that the measurement height noise of the rough surface should be less than the half wavelength used in fine surface measurement. If the measurement noise is not enough to be reduced, the phase jumps cannot be fully compensated for, as seen in Figure 5f. Therefore, the visibility of the interference fringe should be precisely measured for the purpose and the following issues should be carefully considered in addition to the typical considerations of multi-wavelength interferometry:

— Coherent noise reduction and parasitic interference fringe;
— Preliminary determination of fringe visibilities at *λ*_1_ and *λ*_2_.

In fundamental, the visibility is independent on the background intensity, but the coherent noise and intensity variation practically affect to the visibility as noise sources. In particular, diffraction patterns can increase the fluctuation of visibility and should be removed. In this investigation, a speckle reducer (SR) was used to reduce the coherent noise in the system, but its operating time resulted in increasing the exposure time of PCAM, which induced vibration noise in the measurement. Because of this vibration noise, the parasitic interference fringe was shown in the visibility and it restricted obtaining a more precise rough phase map. In order to eliminate the vibration noise, the exposure time of the PCAM should be minimized and the calculated visibility should be calibrated with the interference fringe pattern.

Secondly, the proposed system needs preliminary information about the visibilities at *λ*_1_ and *λ*_2_. The visibility is determined by the amplitude ratio between the reference and measurement waves, the alignment status and the temporal coherence of the laser source. These parameters can be theoretically and experimentally obtained if the material information and the wavelength of the laser light are provided. If the type of measurement targets is changed, preliminary work to know the visibility should be taken again but it can be applied to the same target type after that. 

## 5. Conclusions

In this investigation, we described the effective single shot spatial phase shifting interferometry. Based on two-wavelength interferometry with a polarization pixelated camera (PCAM), the proposed technique has the ability to obtain the 3D surface profile of the specimen at once without any significant lateral resolution reduction and chromatic features of the optical system. To verify the measurement principle of the proposed system, feasibility tests were performed with a step-height specimen and some considerable issues were discussed.
